# Supplementary material for: Human immunocompetent choroid-on-chip: a novel tool for studying ocular effects of biological drugs
Source: Commun Biol. 2022 Jan 13;5:52. doi: 10.1038/s42003-021-02977-3 (PMC8758775; doi:10.1038/s42003-021-02977-3)
Supplement: Supplementary file 4 — Description of Additional Supplementary Files [file 42003_2021_2977_MOESM4_ESM.pdf]

Description of Additional Supplementary Files

File name: Supplementary Data 1

Description: Contains a cell sheet with all source data for graphs and one cell sheet per graph containing the statistical analysis in detail, including graph with Individual data points
