# Supplementary material for: Human immunocompetent choroid-on-chip: a novel tool for studying ocular effects of biological drugs
Source: Commun Biol. 2022 Jan 13;5:52. doi: 10.1038/s42003-021-02977-3 (PMC8758775; doi:10.1038/s42003-021-02977-3)
Supplement: Supplementary file 6 — Reporting Summary [file 42003_2021_2977_MOESM6_ESM.pdf]

Corresponding author(s): Peter Loskill

Last updated by author(s): Nov 3, 2021

Reporting Summary

Nature Research wishes to improve the reproducibility of the work that we publish. This form provides structure for consistency and transparency in reporting. For further information on Nature Research policies, see our Editorial Policies and the Editorial Policy Checklist.

Statistics

For all statistical analyses, confirm that the following items are present in the figure legend, table legend, main text, or Methods section.

n/a	Confirmed	
☐	☒	The exact sample size (*n*) for each experimental group/condition, given as a discrete number and unit of measurement
☐	☒	A statement on whether measurements were taken from distinct samples or whether the same sample was measured repeatedly
☐	☒	The statistical test(s) used AND whether they are one- or two-sided<br>*Only common tests should be described solely by name; describe more complex techniques in the Methods section.*
☐	☒	A description of all covariates tested
☐	☒	A description of any assumptions or corrections, such as tests of normality and adjustment for multiple comparisons
☐	☒	A full description of the statistical parameters including central tendency (e.g. means) or other basic estimates (e.g. regression coefficient) AND variation (e.g. standard deviation) or associated estimates of uncertainty (e.g. confidence intervals)
☐	☒	For null hypothesis testing, the test statistic (e.g. *F*, *t*, *r*) with confidence intervals, effect sizes, degrees of freedom and *P* value noted<br>*Give P values as exact values whenever suitable.*
☒	☐	For Bayesian analysis, information on the choice of priors and Markov chain Monte Carlo settings
☐	☒	For hierarchical and complex designs, identification of the appropriate level for tests and full reporting of outcomes
☒	☐	Estimates of effect sizes (e.g. Cohen's *d*, Pearson's *r*), indicating how they were calculated

Our web collection on statistics for biologists contains articles on many of the points above.

Software and code

Policy information about availability of computer code

Data collection	Immunofluorecence and Permeability assays - Leica Application Suite X (LAS X)<br>Confocal microscopy - ZEN 2012 SP1 (black edition), Release Version 8.1<br>Flow Cytometry - Guava Incyte software (version 2.7)<br>Viability assays on the Guava - Guava ViaCount (version 2.7)<br>Cytokine analysis - Guava Express Pro (version 2.7)
Data analysis	Cytokine analysis - LEGENDplex™ v.8.0 software<br>Flow Cytometry - Guava Incyte software (version 2.7)<br>Viability assays on the Guava - ViaCount (version 2.7)<br>Permeability assays and 3D image quantification - ImageJ - macros are provided as supplementary material<br>Graph drawing and statistical analysis - GraphPad Prism (version 8.2.0)

For manuscripts utilizing custom algorithms or software that are central to the research but not yet described in published literature, software must be made available to editors and reviewers. We strongly encourage code deposition in a community repository (e.g. GitHub). See the Nature Research guidelines for submitting code & software for further information.

Data

Policy information about availability of data

All manuscripts must include a data availability statement. This statement should provide the following information, where applicable:

- Accession codes, unique identifiers, or web links for publicly available datasets
- A list of figures that have associated raw data
- A description of any restrictions on data availability

The data that support the findings of this study are available from the corresponding author upon reasonable request.

Field-specific reporting

Please select the one below that is the best fit for your research. If you are not sure, read the appropriate sections before making your selection.

☒ Life sciences ☐ Behavioural & social sciences ☐ Ecological, evolutionary & environmental sciences

For a reference copy of the document with all sections, see nature.com/documents/nr-reporting-summary-flat.pdf

Life sciences study design

All studies must disclose on these points even when the disclosure is negative.

Sample size	Every chip is considered an independent biological experiment. For each run of chips, the same condition was performed at least twice. For all graphs with a statistical analysis, the minimum sample size is 3. A minimum of 2 runs and a maximum of 12 runs was performed. The sample size criteria depended on the logistical complexity of the whole experiment, primary cell availability, distribution of donors to assure paired conditions, availability of pumping systems and technical issues. The criteria was the following: for proof of concept data, the sample size is at least of 2; for the validation data (PBMCs vs Act) and for the proof of concept study a minimum of 4 (CsA study and TCB data). Every test was performed in parallel with a respective control (PBMC and Act).
Data exclusions	Chips were excluded and not analyzed if there would be technical issues with the connection and pumping system. Only chips with the correct effluent volume were considered.
Replication	Replication was sucessful. Variability is within biological variabliabily for a complex system with 4 different cell types cultured simultaneusly.
Randomization	Chips were randomly allocated into experimental groups using a numbering system for distinguishing them.
Blinding	The numbering system was kept until analysis.

Reporting for specific materials, systems and methods

We require information from authors about some types of materials, experimental systems and methods used in many studies. Here, indicate whether each material, system or method listed is relevant to your study. If you are not sure if a list item applies to your research, read the appropriate section before selecting a response.

Materials & experimental systems

n/a	Involved in the study	
☐	☒	Antibodies
☒	☐	Eukaryotic cell lines
☒	☐	Palaeontology and archaeology
☒	☐	Animals and other organisms
☒	☐	Human research participants
☒	☐	Clinical data
☒	☐	Dual use research of concern

Methods

n/a	Involved in the study	
☒	☐	ChIP-seq
☐	☒	Flow cytometry
☒	☐	MRI-based neuroimaging

Antibodies

Antibodies used

This information is provided in detail in the methods section, as table.
Table 1. Flow cytometry antibodies and isotype controls
Fluorophore Reactivity Antigen Clone Lot Number Product Reference
APC anti-human CD25 BC96 B258747 302610
FITC anti-human CD69 FN50 B224681 310904
PerCP anti-human CD8a HIT8a B241517 300922
FITC Mouse IgG1,κ MOPC-21 B199152 400107

APC Mouse IgG1,κ MOPC-21 B257953 400119
PerCP Mouse IgG1,κ MOPC-21 B226117 400147

Table 2. Immunofluorescence antibodies
AB Supplier, Reference Host Reactivity
CD-31 (PECAM-1) Dako, M0823 Mouse Human
ZO-1 ThermoFisher, 40-2200 Rabbit Human
TYRP1 Abcam, 3312 Mouse Human
Ki-67 R&D Systems, AF7617 Sheep Human
CD3-FITC Biolegend, 300406 Mouse Human
Alexa Fluor 488 ThermoFisher, A11008 Goat Rabbit
Alexa Fluor 546 ThermoFisher, A11003 Goat Mouse
Alexa Fluor 546 ThermoFisher, A21098 Donkey Sheep

Validation

Positive and negative controls were used for all antibodies as well as secondary antibody controls where applicable. Antibody Concentration was used as recomended by the manufactured for flow cytometry assays. For imaging purposes, the concentration was tested in dilutions of 1:50, 1:100 and 1:500. The highest concentrations were selected considering the background/signal ratio and that, in the chip, the volume to cell number (3D tissue) is significantly lower than in tissue slices or cells cultured as monolayers in well plates.

Flow Cytometry

Plots

Confirm that:

☒ The axis labels state the marker and fluorochrome used (e.g. CD4-FITC).

☒ The axis scales are clearly visible. Include numbers along axes only for bottom left plot of group (a 'group' is an analysis of identical markers).

☒ All plots are contour plots with outliers or pseudocolor plots.

☒ A numerical value for number of cells or percentage (with statistics) is provided.

Methodology

Sample preparation

For flow cytometry analysis cells were washed and labeled with anti-CD8a-PerPC, anti-CD25-APC and anti-CD69-FITC (Biolegend) for 20 min at 4°C. Antibodies were used at concentrations recommended by the manufacturer. As buffer solution and for the washing step PBS containing 2 mM of EDTA and 0.1 % of BSA was used. Autofluorescence and isotype controls were performed to confirm similar levels of unspecific signal in every staining. Table 1 specifies the used antibodies and isotype controls

Instrument

Guava EasyCyte HT (Guava, Luminex)

Software

Guava Incyte software

Cell population abundance

5.8% to 38% abundance

Gating strategy

The PBMCs were gated on the FCS/SSC plot before gating for CD8a. The quantification of the fraction of CD25 and CD69 positive cells was performed for the CD8a positive population.

☒ Tick this box to confirm that a figure exemplifying the gating strategy is provided in the Supplementary Information.
